# Generation and validation of a conditional knockout mouse model for desmosterolosis

**DOI:** 10.1016/j.jlr.2021.100028

**Published:** 2021-01-30

**Authors:** Babunageswararao Kanuri, Vincent Fong, Sithara Raju Ponny, Ranjuna Weerasekera, Kirthi Pulakanti, Kriya S. Patel, Roman Tyshynsky, Shailendra B. Patel

**Affiliations:** 1 Division of Endocrinology, Diabetes and Metabolism, University of Cincinnati, Cincinnati, OH, USA; 2 Division of Human Genetics, Cincinnati Children’s Hospital Medical Center, Cincinnati, OH, USA; 3 Division of Endocrinology, Medical College of Wisconsin, Milwaukee, WI, USA; 4 Blood Research Institute, Versiti, Milwaukee, WI, USA; 5 Clement J. Zablocki Veterans Affairs Medical Center, Milwaukee, WI, USA

**Keywords:** DHCR24, desmosterol, liver, dysmorphology, cholesterol, bile, animal models, gene expression, liver-X-receptor, lipoproteins, 7-DHC, 7-dehydrocholsterol, CD36, cluster of differentiation 36, CEACAM1, carcinoembryonic antigen-related cell adhesion molecule 1, CYP7A1, cytochrome P450 family 7 subfamily A member 1, CYP27A1, cytochrome P450 family 27 subfamily A member 1, CYP8B1, cytochrome P450 family 8 subfamily B member 1, DHCR7, 3β-hydroxysterol-Δ7 reductase, DHCR24, 3β-hydroxysterol-Δ24 reductase, FAS, fatty acid synthase, FDFT1, farnesyl-diphosphate farnesyl transferase 1, GEO, Gene Expression Omnibus, HMGCR, 3-hydroxy-3-methylglutaryl-CoA reductase, LDLR, low density lipoprotein receptor, LIPC, hepatic triacylglycerol lipase, LXR, liver X receptor, MTTP, microsomal triglyceride transfer protein, NR1H2/3/4, nuclear receptor subfamily 1 group H member 2/3/4, PCSK9, proprotein convertase subtilisin/kexin type 9, SCARB1, Scavenger receptor class B member 1, SREBF1/2, sterol regulatory element binding transcription factor 1/2

## Abstract

The enzyme 3β-hydroxysterol-Δ24 reductase (DHCR24, EC 1.3.1.72) catalyzes the conversion of desmosterol to cholesterol and is obligatory for post-squalene cholesterol synthesis. Genetic loss of this enzyme results in desmosterolosis (MIM #602398), a rare disease that presents with multiple congenital anomalies, features of which overlap with subjects with the Smith-Lemli-Opitz syndrome (another post-squalene cholesterol disorder). Global knockout (KO) of *Dhcr24* in mice recapitulates the biochemical phenotype, but pups die within 24 h from a lethal dermopathy, limiting its utility as a disease model. Here, we report a conditional KO mouse model (*Dhcr24*^flx/flx^) and validate it by generating a liver-specific KO (*Dhcr24*^flx/flx,Alb-Cre^). *Dhcr24*^flx/flx,Alb-Cre^ mice showed normal growth and fertility, while accumulating significantly elevated levels of desmosterol in plasma and liver. Of interest, despite the loss of cholesterol synthesis in the liver, hepatic architecture, gene expression of sterol synthesis genes, and lipoprotein secretion appeared unchanged. The increased desmosterol content in bile and stool indicated a possible compensatory role of hepatobiliary secretion in maintaining sterol homeostasis. This mouse model should now allow for the study of the effects of postnatal loss of DHCR24, as well as role of tissue-specific loss of this enzyme during development and adulthood.

In cholesterol biosynthesis, the conversion of lanosterol to cholesterol has been described to occur via two main pathways, the Bloch pathway (1) and the Kandutsch-Russell pathway (2), which differ in whether reduction of the side chain C24-25 double bond occurs “first” or “last.” In the Kandutsch-Russell pathway the C24-C25 reduction takes place in the first step, whereas in the Bloch pathway this reduction occurs in the last step of the synthesis. This reaction is catalyzed by 3β-hydroxysterol Δ24-reductase (DHCR24, EC 1.3.1.72) and is required for cholesterol biosynthesis (3). Most commonly described as the enzyme catalyzing the conversion of desmosterol to cholesterol, DHCR24 can reduce the C24-25 double bond of any sterol intermediate, effectively switching sterol synthesis from the Bloch pathway to Kandutsch-Russell pathway. This hybrid or modified Kandutsch-Russell pathway is likely how many (if not all) mammalian cells synthesize cholesterol, rather than by using one pathway exclusively (4, 5), and modifies the concept of first or last step. Alterations in DHCR24 activity lead to the accumulation of different biosynthetic intermediates (5), which have distinct biological activities and effects on downstream pathways (6). Variations in DHCR24 expression have been linked to different human diseases, including Alzheimer’s disease, cardiovascular disease, hepatitis C virus infection, and prostate cancer (3), although none of these have been rigorously tested.

Homozygous or compound heterozygous defects in two terminal enzymes participating in cholesterol synthesis, DHCR7 or DHCR24, cause the dysmorphological conditions Smith-Lemli-Opitz syndrome (OMIM #270400) and desmosterolosis (OMIM #602398), respectively (7, 8). Although endogenous cholesterol biosynthesis is lost, the affected individuals are still exposed to some cholesterol via maternal-fetal transfer in utero and subsequently from dietary sources after birth (9). Desmosterolosis is a very rare disease, with only nine published cases, and has an estimated incidence of <1:10,000,000, compared with Smith-Lemli-Opitz syndrome with an observed incidence of 1:20,000 to 60,000 (6, 10, 11). Patients with desmosterolosis manifest a spectrum of defects affecting craniofacial and neurological development, intellectual disability, and developmental delays, with the most severe cases dying shortly after birth (8, 12, 13, 14, 15, 16). Although the initial publication describing *Dhcr24* knockout (KO) mice reported survival of KO into adulthood (17), this knockout line was freely shared with several investigators and all subsequent knockout progeny failed to survive beyond 24 h post birth as a result of a lethal dermopathy (18), a finding confirmed by the original investigators (19). Therefore, the absence of a viable animal model for desmosterolosis limits exploration of the underlying pathological mechanisms resulting from loss of DHCR24 and also prevents the exploration of mechanisms testing the effects of desmosterol in mammalian physiology. We sought to generate a viable mouse model with tissue-specific deletion of *Dhcr24* to avoid the lethal dermopathy. In this study, we describe the characterization of a conditional KO of *Dhcr24* using the Cre-loxP system and validated this model by creating a liver-specific loss of *Dhcr24*.

## Methods

### Generation of KO mice

**Fig. 1:**
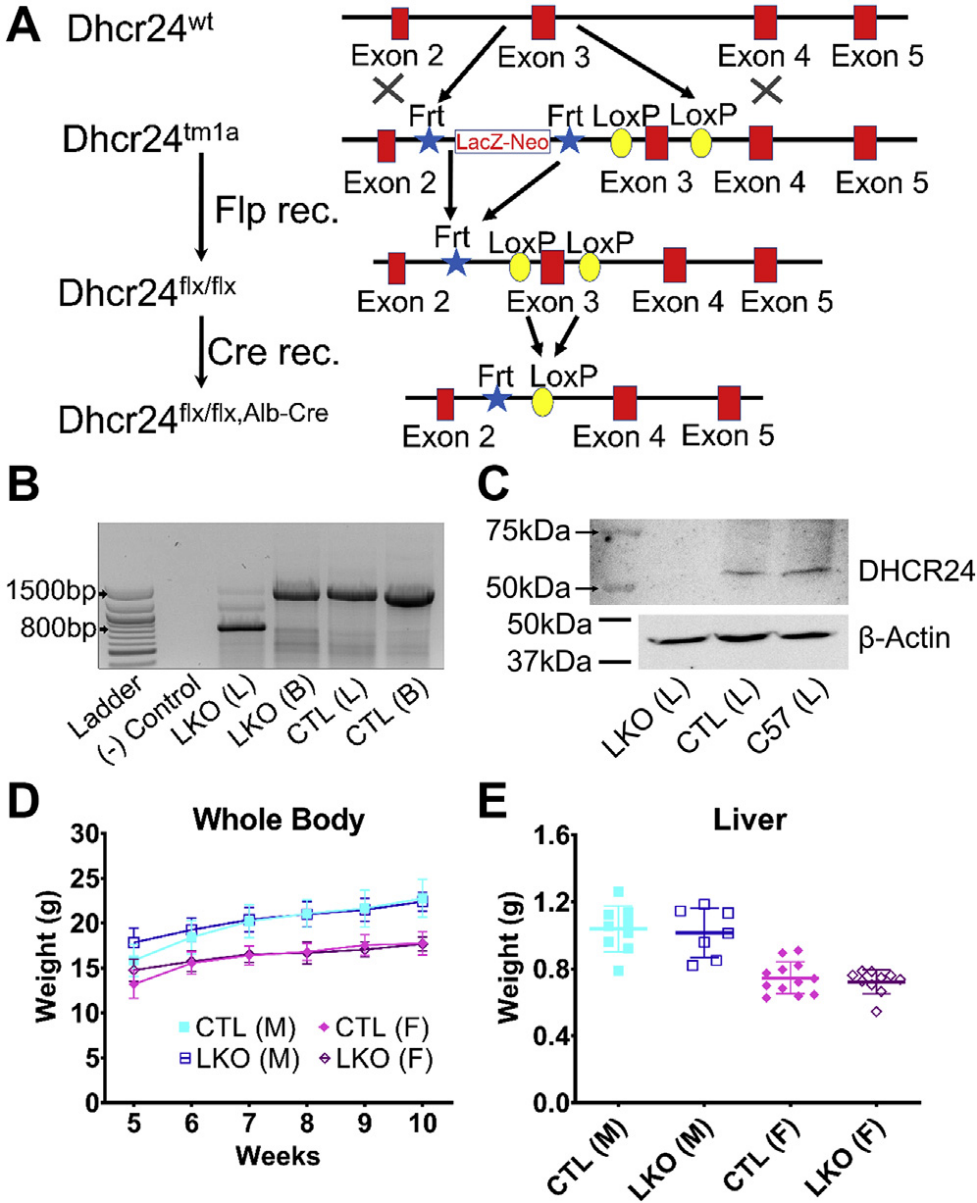
Verification of liver-specific deletion of *Dhcr24* gene and growth curves in *Dhcr24*^flx/flx,Alb-Cre^ mice. A schematic representation of the targeting strategy used for generation of *Dhcr24* conditional KO mice (*Dhcr24*^flx/flx^) and hepatocyte-specific deletion (*Dhcr24*^flx/flx,Alb-Cre^, A). PCR of genomic DNA isolated from liver (L) and brain (B) confirms hepatocyte-specific deletion of exon 3 (B). The faint band corresponding to WT exon 3 in the LKO liver lane is likely from nonhepatocyte cells in the liver. Western blot analysis shows the bona fide loss of DHCR24 protein expression in LKO liver (C). The growth velocity of both male (M) and female (F) mice measured from 5 to 10 weeks (wk) of age was comparable between liver-specific knockout (*Dhcr24*^flx/flx,Alb-Cre^, LKO) and control mice (*Dhcr24*^flx/flx^, CTL) (D); for male LKO mice N = 13 in wk 5–8, N = 9 in wk 9, and N = 10 in wk 8; for male CTL mice N = 8 in wk 5, N = 12 in wk 6, N = 15 in wk 7–9, and N = 9 in wk 10; for female LKO mice, N = 17 in wk 5–8, N = 15 in wk 9, and N = 11 in wk 10; for female CTL mice, N = 9 in wk 5, N = 13 in wk 6, N = 18 in wk 7, N = 17 in wk 8–9, and N = 12 in wk 10. There were no differences in liver weights between LKO and CTL mice sacrificed at 10–12 weeks of age (E); N = 9 male CTL, N = 7 male LKO, N = 12 female CTL, and N = 10 female LKO mice. Males are represented with squares, and females with diamonds; closed symbols indicate CTL mice, and open symbols LKO mice. Bars denote mean ± 1 SD.

All animal protocols were approved initially by the Medical College of Wisconsin, Milwaukee, WI, and the Clement J Zablocki Veterans Affairs (VA) IACUC, Milwaukee, WI, and subsequently by the University of Cincinnati IACUC, Cincinnati, OH. Live mice, harboring a floxed *Dhcr24* allele (*Dhcr24*^tm1a(EUCOMM)Wtsi^), were imported from the Wellcome Trust Sanger Center, via EUCOMM (20, 21, 22, 23). Subsequent breeding and germline transmission were confirmed using PCR but showed that the LacZ-Neo cassette (Fig. 1A) was interfering with normal *Dhcr24* expression as no homozygous mice were observed from heterozygous matings. The lacZ-Neo cassette (Fig. 1A) was removed by breeding with mice expressing FLP recombinase obtained from Jackson laboratories (B6.129S4-*Gt(ROSA)26Sor*^*tm1(FLP1)Dym*^/RainJ; #009086). Subsequent breeding resulted in a viable *Dhcr24*^flx/flx^ line (also referred to as CTL, for control), and these mice were normal and fertile, with no elevations of plasma desmosterol, compared with wild-type littermates. Mice heterozygous for the floxed allele were backcrossed for five generations to C57Bl/6J before crossing with Alb-cre mice. Liver-specific KO mice (LKO) were generated by mating with mice expressing Cre recombinase driven by albumin promoter obtained from Jackson laboratories [B6.Cg-*Speer6-ps1*^*Tg(Alb-cre)21Mgn*^/J; #003574] and are referred to as *Dhcr24*^flx/flx,Alb-Cre^ (also referred to as LKO, for liver knockout). For one set of experiments, adult mice with postnatal global loss of *Dhcr24* were generated by breeding *Dhcr24*^flx/flx^ mice with tamoxifen-inducible ER-Cre strain [B6;129-*Gt(ROSA)26Sor*^*tm1(cre/ERT)Nat*^/J; #004847] and are referred to as *Dhcr24*^flx/flx,Er-Cre^. Animal lines were maintained on a regular chow diet (Envigo 7912; Harlan Teklad, Madison, WI) and housed in individually ventilated PIV cages. To induce postnatal global loss of Dhcr24, *Dhcr24*^flx/flx,Er-Cre^ mice were intercrossed and progeny injected at 4–5 weeks of age with tamoxifen (T5648; Sigma-Aldrich, St. Louis, MO) dissolved in canola oil (10 mg/ml) at 1 mg per mouse or vehicle (time 0) and monitored weekly.

### Genotyping details

Genotypes of *Dhcr24*^flx/flx,Alb-Cre^ mice, *Dhcr24*^flx/flx,Er-Cre^ mice, and their respective controls were determined by evaluating homozygosity of flox alleles and the presence of Cre based on PCR of tail snip genomic DNA. The presence of the floxed allele was determined by amplification of a 652 bp fragment using primers Dhcr24-flp-for (5’-caaagcatacgaaagagcagcac-3’) and Dhcr24-3’arm (5’-tcaagctcaggcaacacaggcagg-3’), compared with the wild-type allele of 442 bp fragment using these primers. The presence of Cre was detected through amplification of a 408 bp fragment using 5’-gcattaccggtcgatgcaacgagtgatgag-3’ and 5’-gagtgaacgaacctggtcgaaatcagtgcg-3’ primers. The presence or absence of exon 3 was detected with primer Dhcr24-flp-for (5’-caaagcatacgaaagagcagcac-3’) and primer Dhcr24 cre confirm-rev (5’-agctcgtaggcagtgcaaat-3’) PCR products (intact floxed allele product of 1,457 bp compared with Exon 3 deletion after cre-mediated recombination of 801 bp) of the genomic DNA isolated from tissues by running on a 1.5% agarose gel under standard conditions using a 100 bp DNA ladder for identification (see also supplemental Fig. S1). DNA was stained using SYBR Safe DNA gel stain (S33102, ThermoFisher Scientific, Waltham, MA) and scanned on a gel station (Universal Hood II, Bio-Rad). Protein expression was assessed by Western blot analysis of liver tissue lysate and probing with anti-DHCR24 Antibody (C59D8; Cell Signaling, Danvers, MA).

### Plasma, bile, and stool collection

Four-week-old postweaned mice were recruited for weekly body weight measurements. Blood draws were from the submandibular vein. For bile measurements, mice were weighed and, under isoflurane anesthesia, the gallbladder was cannulated after the common bile duct had been ligated and timed bile flow under gravity was determined (24). We maintained a standard 4 h fast period while performing plasma and bile isolation experiments. For stool collection, mice were singly housed in clean cages and fecal pellets were collected and stored at −80°C for subsequent assays.

### Tissue harvesting

Mice 10–12 weeks of age were euthanized under CO_2_ anesthesia; liver and brain tissues were collected and immediately flash frozen in liquid N_2_ and stored at −80°C for subsequent estimation of tissue sterols or RNA isolations. For histology, a small portion of liver from male CTL and LKO mice aged 12 weeks was rinsed twice in ice-cold phosphate buffered saline (PBS; pH7.4) and fixed overnight in 10% neutral buffered formalin (5725; Fisher Scientific, Pittsburgh, PA) followed by embedding in paraffin for hematoxylin and eosin staining or directly embedded in OCT compound for oil red O staining (21). Hepatic triglyceride secretion rates were determined, after 4 h of fasting and at ∼11 AM, by collection of blood at 0, 30, 60, 90, 120 min after intraperitoneal injection of poloxamer 407 (P-407, a.k.a. Pluronic® F127) at 1 g/kg dose (P2443; Sigma-Aldrich, St. Louis, MO) (25).

### Sterols estimation using gas chromatography/mass spectrometry

Sterol analyses of plasma, bile, liver, brain, heart, and feces were measured using capillary column gas chromatography-mass spectrometry (GC/MS) as described previously (21, 24). Briefly, post hexane extraction and trimethylsilyl derivatization, test samples were injected into a 30 m low polarity phase crossbond diphenyl dimethyl polysiloxane Restek-5ms column (Restek Cat No. 13423) on a Thermo-Finnegan FOCUS GC/MS (ThermoFisher) measuring the intensities of the 329, 343, 393, 255, and 325 *m/z* peaks for cholesterol, desmosterol, lanosterol, lathosterol, and 7-DHC, respectively (26). The retention times of different sterols were verified by matching the retention times of the derivatives of the known standard compounds. A known amount of 5α-cholestane was added as an internal standard (24).

### Fast-protein liquid chromatography

Lipoprotein separation for total cholesterol and triglycerides estimation in plasma samples was performed using fast-protein liquid chromatography (FPLC) as described elsewhere (27). Briefly, mice were fasted 4 h, and blood was collected in anticoagulant (EDTA)-coated tubes through the submandibular plexus. Samples were centrifuged at 2500 *g* for 10 min at 4°C, and plasma was collected and the total cholesterol was measured (using an enzymatic kit) to ensure that pooling of samples used plasma with comparable sterols to exclude any outliers with any dramatic differences between individual mice (although no samples needed to be excluded). Column chromatography (10/300GL, Superose 6) was then used for separation of different lipoproteins by loading a minimum of 100 μl of pooled plasma mixed with 100 μl of PBS, from each group, onto the Akta pure FPLC, and a total of 52 fractions each with volume of 500 μl were collected and analyzed for cholesterol content using Infinity cholesterol (TR13421) and triglyceride (TR22421) calorimetric kits procured from ThermoFisher Scientific, Middletown, VA.

### RNA isolation and qPCR assessments

Total RNA was isolated from liver and spleen tissues (n = 4 per group) by the column purification method using a Qiagen RNeasy® Kit (74104; Qiagen Inc., Germantown, MD) and was reverse transcribed to cDNA using the High-Capacity RNA-to-cDNA™ Kit (4387406; ABI, Foster City, CA). Quantitative assessment of *Nr1h3* and LXR target genes was performed on Applied Biosystems 7300 Real-Time PCR system (Applied Biosystems, Foster City, CA) using commercially available Taqman real-time PCR probes (ABI Biosystems). All reactions were performed in triplicate, and *Atp5po* was used as a housekeeping gene for data normalization to compensate the variations between input RNA amounts and analyzed using the comparative C_T_ method (21).

### RNA-Seq experimental design

RNA-Seq experiments were performed on total RNA isolated from liver and adrenal glands, using the total RNA isolated by the RNeasy Mini kit column method (74104; Qiagen, Valencia, CA). Total RNA from three female mice for each group was pooled together into a single test and control sample for library preparation followed by sequencing. RNA-Seq libraries were prepared using the NEBNext Ultra Directional RNA Library Prep Kit (New England BioLabs, Ipswich, MA) and sequenced using the TruSeq SBS kit on HiSeq platform (Illumina, San Diego, CA) at UC Genomics, Epigenomics and Sequencing Core. FASTQ files thus generated were used for bioinformatics analysis via the DNA Sequencing and Genotyping Core Facility of Cincinnati Children’s Hospital Medical Center. In brief, quality control steps were performed on the FASTQ files to determine the overall quality of the reads using FASTQC. FASTQ files were then trimmed to remove adapter sequences and low-quality reads using Trimmomatic. The trimmed reads were then mapped to the mouse (mm10) reference genome using Hisat2. In the next step, transcript/gene abundance was assessed using Kallisto by creating a transcriptome index for Ensemble cDNA sequences from mouse (mm10). This index was used to quantify transcript abundance in raw counts and transcript per million. The raw count matrix from Kallisto was then used to identify differentially expressed genes between *Dhcr24*^flx/flx,Alb-Cre^ and *Dhcr24*^flx/flx^ using RUVSeq (R package). Differentially expressed genes showing statistical significance were defined using two filters: fold change cutoff of 2 and adjusted *P*-value/*P*-value cutoff of ≤ 0.05. Downstream functional annotation of significantly dysregulated genes was determined using gene ontology (cellular components, molecular function, and biological process) and pathway analysis. A detailed functional annotation and pathway analysis was performed using TOPPFUN part of the TOPPGENE suite (28).

### Statistical analyses

RNA-Seq data were analyzed as described above. All other data are shown as mean ± SD. Data were assessed using Shapiro-Wilk’s/Kolmogorov-Smirnov/D’Agostino and Pearson omnibus normality tests to determine whether it followed a Gaussian distribution prior to analyses. Statistical significance of differences between groups was evaluated by independent Student’s *t*-test or Mann-Whitney *U* test or multiple *t*-test where applicable. A variation with *P* ≤ 0.05 was considered statistically significant.

## Results

### Verification of liver-specific deletion of *Dhcr24*

The strategy employed for creating the liver-specific knockout mice of *Dhcr24* gene is detailed in Fig. 1A. To verify that there was liver-specific deletion of *Dhcr24*, we performed PCR and Western blot analyses. PCR using genomic DNA isolated from liver of *Dhcr24*^flx/flx,Alb-Cre^ mice (LKO, Fig. 1) showed a smaller product than that of liver of *Dhcr24*^flx/flx^ mice (CTL, Fig. 1), indicating deletion of the *Dhcr24* alleles (Fig. 1B). However, PCR products of DNA from the brain of *Dhcr24*^flx/flx,Alb-Cre^ and *Dhcr24*^flx/flx^ mice were not different, thus confirming the liver-specific deletion of *Dhcr24* (Fig. 1B). Western blots confirmed the expression of DHCR24 protein in tissue lysates from livers of *Dhcr24*^flx/flx^ mice but not in livers of *Dhcr24*^flx/flx,Alb/Cre^ mice (Fig. 1C). *Dhcr24*^flx/flx,Alb-Cre^ showed normal growth curves compared with *Dhcr24*^flx/flx^ mice (Fig. 1D) and exhibited normal terminal liver and spleen weights (Fig. 1E and supplemental Fig. S2A).

### *Dhcr24*^flx/flx, Alb-Cre^ mice have elevated liver and plasma levels of desmosterol

**Fig. 2:**
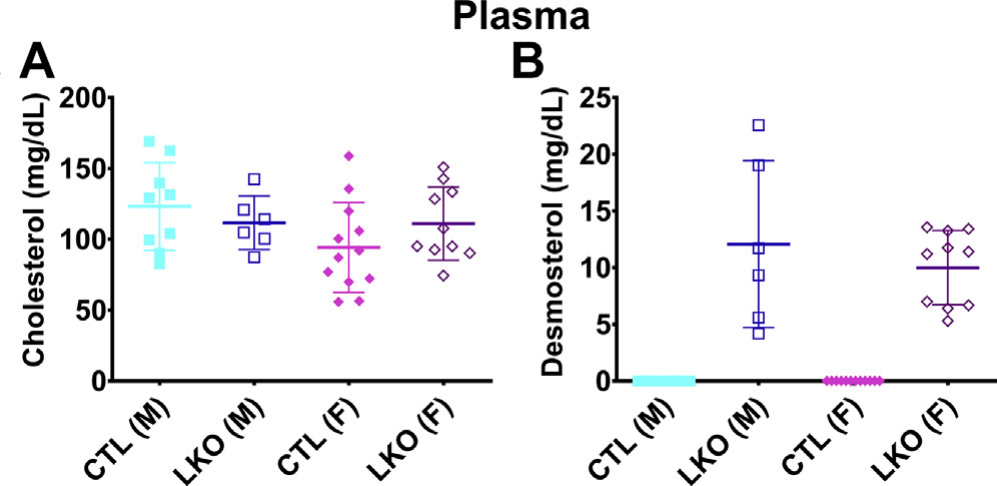
Biochemical characterization of loss of hepatic DHCR24 protein. Plasma cholesterol (A) and desmosterol (B) levels in CTL and LKO mice measured at 9 weeks of age are shown. Cholesterol levels were not significantly different, but desmosterol was dramatically increased in LKO mice, whereas it was below detectable limits in CTL mice. N = 9 male CTL, N = 6 male LKO, N = 12 female CTL, and N = 10 female LKO mice. Males are represented with squares, and females with diamonds; closed symbols indicate CTL mice, and open symbols LKO mice. Bars denote mean ± 1 SD.

**Fig. 3:**
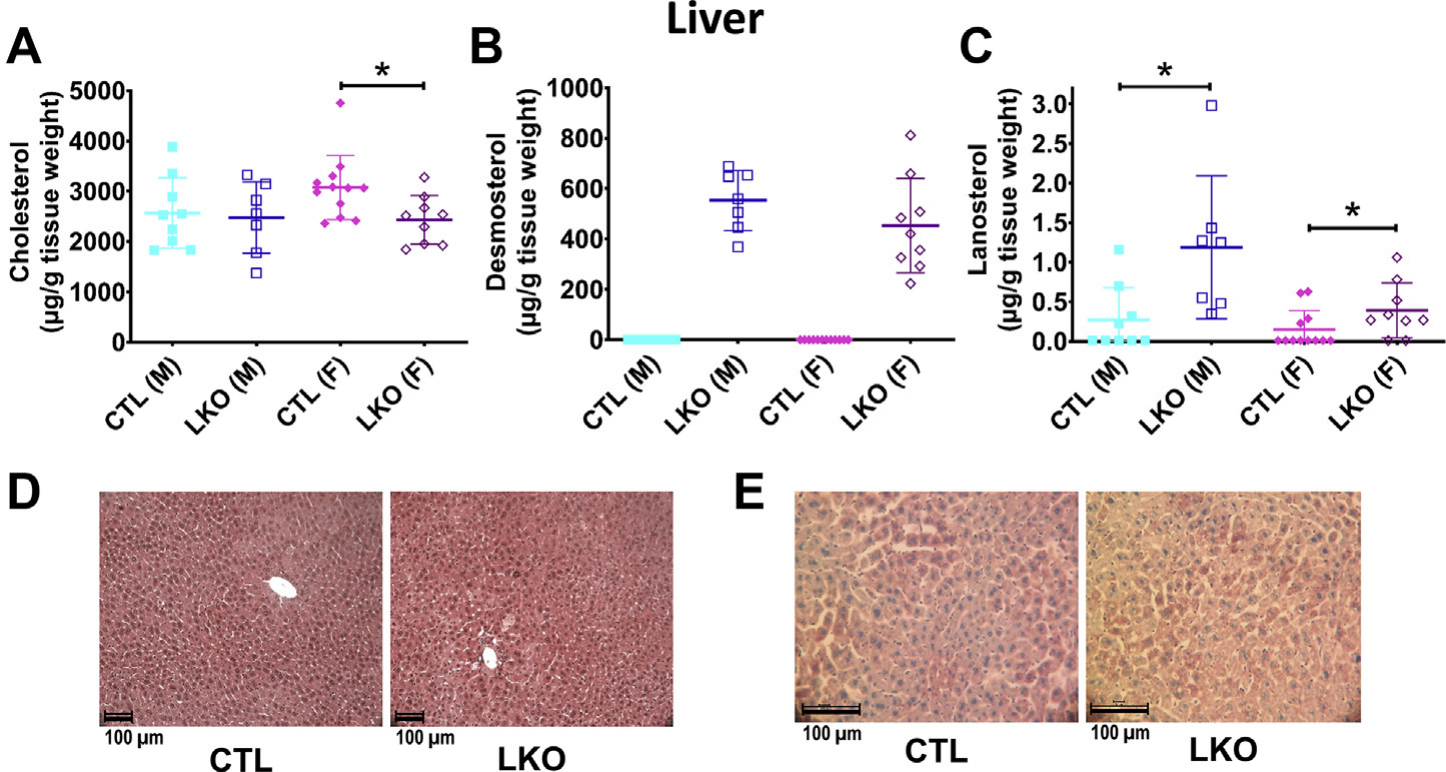
Effect of hepatocyte-specific deletion of *Dhcr24* on hepatic sterol content and liver architecture Hepatic cholesterol (A), desmosterol (B), and lanosterol (C) in CTL and LKO mice are shown. Cholesterol levels in livers of female LKO mice (N = 9) were slightly lower than of female CTL mice (N = 11), while the difference was not significant in males (N = 9 male CTL, N = 7 male LKO, A). Desmosterol levels were markedly higher in livers of male LKO mice (B), while lanosterol was also increased in livers of LKO mice, but with variability among samples. Representative liver sections stained with H&E (D) or oil red O (E) are shown. Liver microarchitecture did not appear to be altered by hepatocyte-specific deletion of *Dhcr24*. Livers were collected from mice euthanized at age 10–12 weeks. Males are represented with squares, and females with diamonds; closed symbols indicate CTL mice, and open symbols LKO mice. Bars denote mean ± 1 SD. (∗*P* < 0.05) Comparisons were performed only with sex-matched controls.

There was no difference in the circulating levels of cholesterol (Fig. 2A) between LKO mice and their sex-matched controls. Plasma desmosterol levels were considerably elevated in LKO mice, whereas desmosterol levels remained undetectable in control mice (Fig. 2B). Levels of hepatic cholesterol (Fig. 3A) were slightly decreased in female *Dhcr24*^flx/flx,Alb-Cre^ mice. The difference was small and was not significant in male mice. Levels of hepatic desmosterol (Fig. 3B) were very elevated, and lanosterol levels slightly elevated (Fig. 3C), in *Dhcr24*^flx/flx,Alb-Cre^ compared with *Dhcr24*^flx/flx^ mice. There were no obvious differences in liver cell morphology or hepatic lipid accumulation (Fig. 3D, E).

Sterol levels in the brains of *Dhcr24*^flx/flx,Alb-Cre^ mice showed no significant differences in the levels of cholesterol (supplemental Fig. S3A), or desmosterol (supplemental Fig. S3B), when compared with *Dhcr24*^flx/flx^ mice, showing that plasma desmosterol did not readily cross the blood-brain barrier and accumulate in this organ.

### Hepatobiliary excretion of desmosterol in *Dhcr24*^flx/flx,Alb-Cre^ mice

**Fig. 4:**
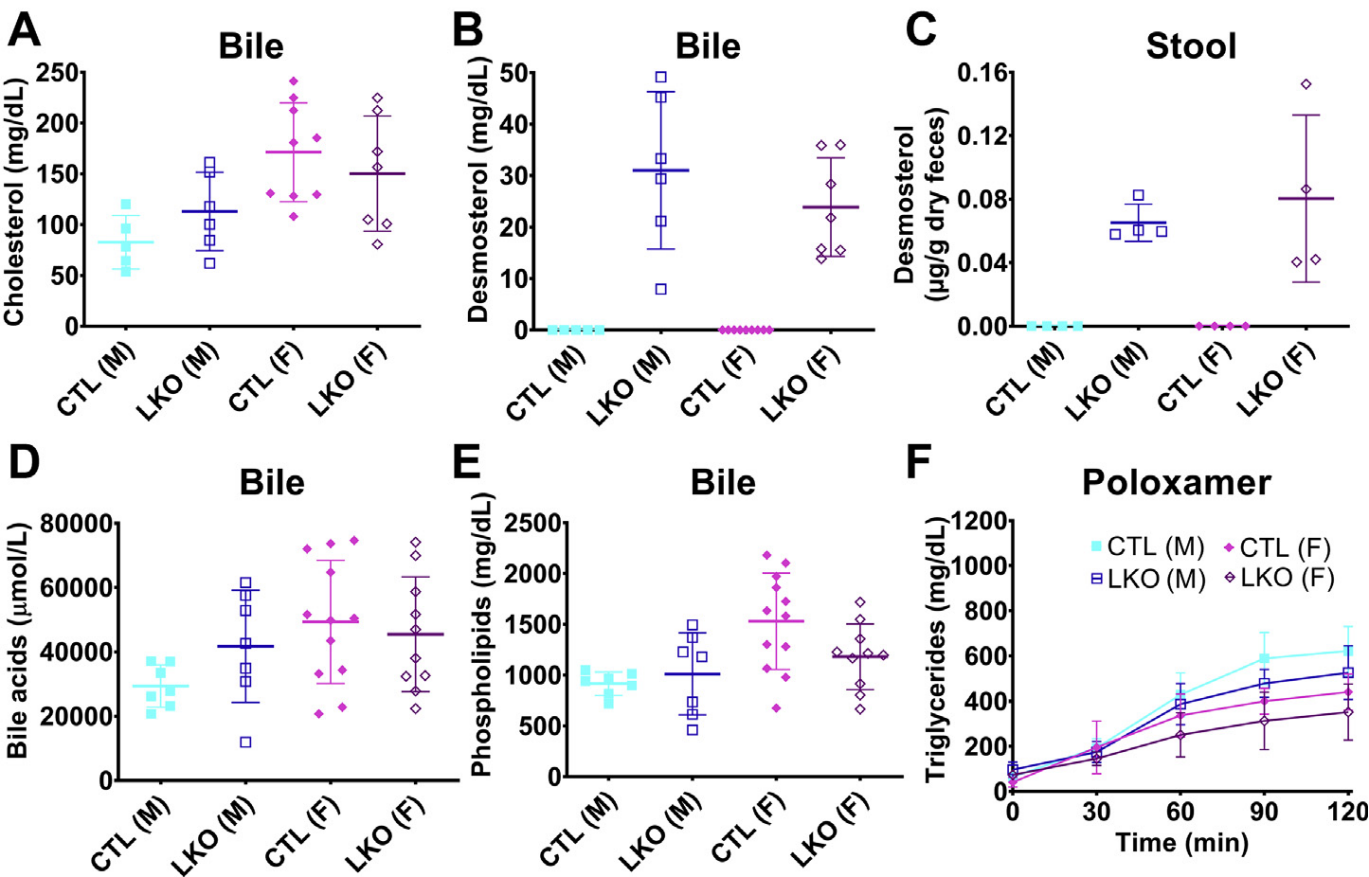
**Effect of hepatic*Dhcr24* deletion on bile components, stool desmosterol, and hepatic triglyceride secretion rates.** Biliary cholesterol (panel A), desmosterol (panel B), total bile acid (panel D) and phospholipid (panel E) levels, stool desmosterol level (panel C), and hepatic triglyceride secretion after IP injection with 1g/kg Poloxamer-407 (panel F) are shown. Although biliary cholesterol was not significantly different between LKO mice and their sex-matched controls (panel A), there was markedly increased excretion of desmosterol into bile and stool of LKO mice (panels B and C), while levels were undetectable in CTL mice. No differences were observed in biliary total bile acid or biliary phospholipid levels (panels D and E). Liver triglyceride secretion rates were not significantly different between LKO and CTL mice (panel F). For bile cholesterol and desmosterol measurements, N=5 male CTL, N=6 male LKO, N=9 female CTL, and N=7 female LKO mice. For bile total bile acid and phospholipid assays, N= 7 male CTL, N=7 male LKO, N=12 female CTL, and N=10 female LKO mice. Bile was collected from mice euthanized at age 10-12wk. For stool desmosterol measurements, N=4 for all groups. For poloxamer assay to assess triglyceride secretion, N=6 male CTL,N=5 male LKO, N=6 female CTL, and N=6 female LKO mice. Males are represented with squares, and females with diamonds; closed symbols indicate CTL mice, and open symbols LKO mice. Bars denote mean ± 1SD. Comparisons were performed only to sex-match controls.

There were no marked differences in the levels of biliary cholesterol, bile acids, and biliary phospholipids (Fig. 4A, D, E) between LKO mice and controls, suggesting only a minimal effect on bile synthesis (29). We hypothesized that hepatobiliary secretion may play a role in disposing of excess desmosterol to maintain sterol homeostasis in *Dhcr24*^flx/flx,Alb-Cre^ mice. Although desmosterol was not detectable in bile or stool from control mice, biliary (Fig. 4B) and stool desmosterol (Fig. 4C) levels were significantly increased in *Dhcr24*^flx/flx,Alb-Cre^ mice, suggesting a possible route for excess desmosterol excretion. There were no changes in the levels of stool cholesterol (supplemental Fig. S3C). Similarly, hepatic triglyceride secretion rates (Fig. 4F) and FPLC cholesterol (supplemental Fig. S4A) and triglyceride lipoprotein profiles (supplemental Fig. S4B) were not significantly different between *Dhcr24*^flx/flx,Alb-Cre^ and *Dhcr24*^flx/flx^ mice.

### Expression of *Nr1h3* and LXR target genes

**Fig. 5:**
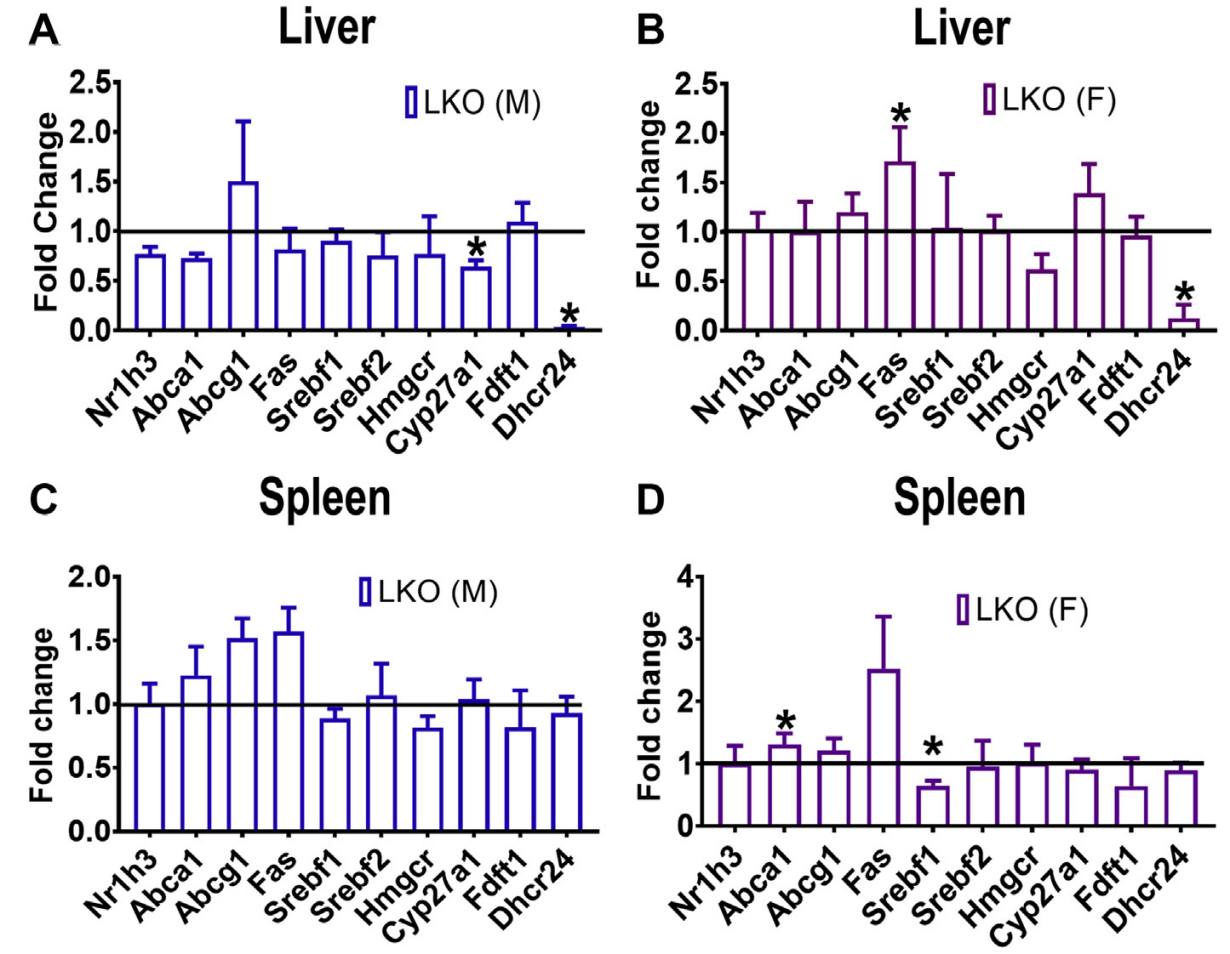
Expression of *Nr1h3*, selected LXR target genes, and sterol synthesis pathway genes. Relative expression of *Nr1h3* and LXR target genes in liver (males: A, females: B) and spleen (males: C, females: D) assessed by qPCR (N = 4) of mice at 10–12 weeks of age are displayed as the fold-change (ratio of LKO/CTL). Deletion of *Dhcr24* in liver with resulting elevated circulatory desmosterol did not have an apparent effect on *Nr1h3* expression in both male and female *Dhcr24*^flx/flx,Alb-Cre^ mice. Error bars denote +1 SD. (∗*P* < 0.05).

Desmosterol is a known activator of the Liver X receptor (LXR), encoded by *Nr1h3*, and has been reported to play a role in foam cell formation (6). Gene expression in liver and spleen were analyzed through qPCR as they express LXR and its target genes, *Abca1*, *Abcg1*, *Fas*, and *Srebf1* (30). It is surprising that no consistent difference in the expression of these genes was found between KO and control mice livers (Fig. 5A, B) and spleens (Fig. 5C, D).

### Gene ontology and pathway analysis

**Fig. 6:**
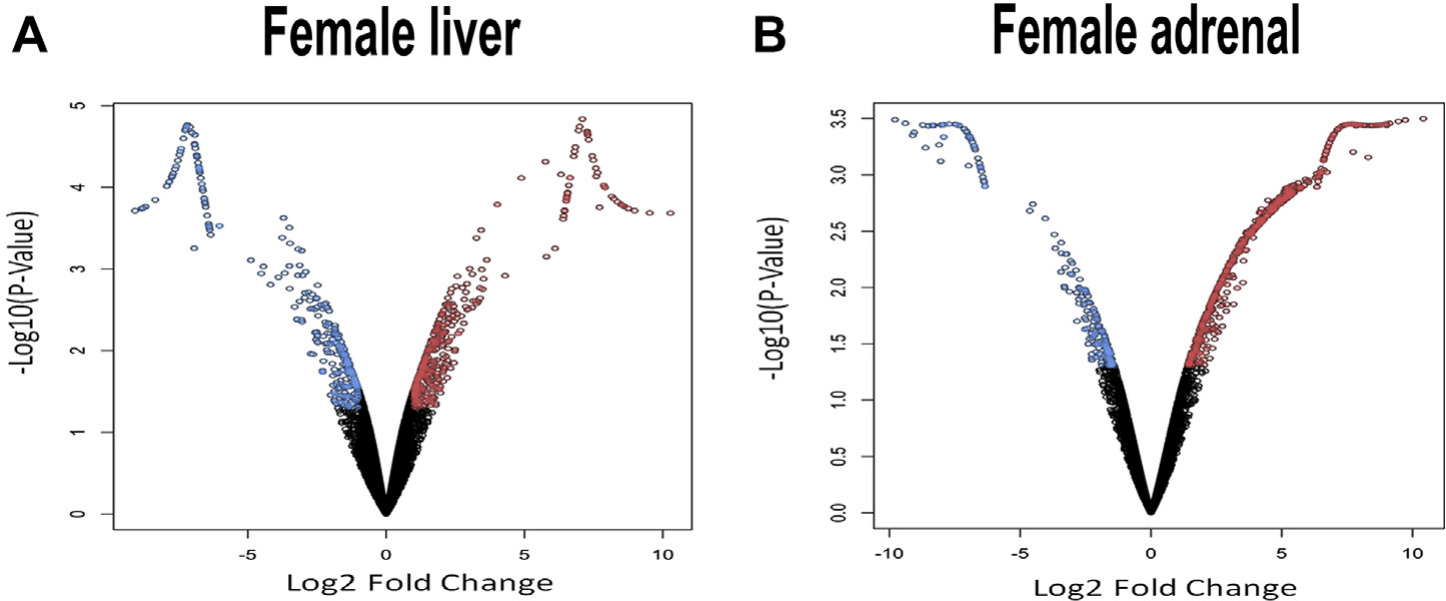
RNA-Seq analysis of female liver and adrenal tissues. Volcano plots (A and B) demonstrate the overall pattern of differential gene expression in female liver and adrenal glands collected at 10–12 weeks of age. Red, blue, and black color in each volcano plot corresponds to the genes significantly upregulated, downregulated, and unchanged in LKO compared with CTL mice of same sex.

Given the changes in the sterol profile of *Dhcr24*^flx/flx,Alb-Cre^ mice, we sought to characterize the broader effects on the differentially regulated transcriptome using RNA-Seq (Fig. 6). We identified 717 differentially expressed genes in the livers of female mice (Fig. 6A). Further analysis revealed association of the differentially expressed genes into 3 upregulated and 16 downregulated pathways. It is surprising that the pathways involved in steroidogenesis and steroid hormone metabolism were enriched by significantly downregulated genes (supplemental Table S1). Notably missing was any major perturbation in the cholesterol synthesis and metabolism pathways. To examine if elevated desmosterol in the blood affected adrenals (a major steroidogenic organ), we performed RNA-Seq on adrenals from *Dhcr24*^flx/flx,Alb-Cre^ mice and compared with controls (*Dhcr24*^flx/flx^ mice, Fig. 6B). Enrichment of differentially expressed genes in steroid hormone biosynthesis was also noted, but interestingly, the enrichment was through significantly upregulated genes instead of downregulation (supplemental Tables S1 and S2). Top hits of pathways enriched by significantly upregulated and downregulated genes are listed in supplemental Tables S1 and S2, and the complete dataset can be found in NCBI Gene Expression Omnibus (GEO) database (#GSE146524). Pathway analyses failed to show a common theme altered by loss of *Dhcr24*. A list of the changes in expression of key genes involved in lipid and cholesterol homeostasis is shown in Table 1. Differences were considered significant based on two criteria: logFC ≥ 1 and FDR ≤ 0.05, with up- or downregulated genes highlighted in bold.

**Table 1:** Relative changes in 30 key genes associated with lipid phenotype in *Dhcr24*^flx/flx,Alb-Cre^ mice

No.	Gene	Female Liver (logFC)	*P*-Value	Female Adrenals (logFC)	*P* Value
	Enzymes involved in cholesterol synthesis				
1	Dhcr7	−0.0093	0.9713	0.0557	0.9117
2	Dhcr24	−**4.1800**	**0.0015**	−0.2607	0.6116
3	Fas	−0.1210	0.6692	0.2124	0.6862
4	Hmgcr	−0.0249	0.9227	−0.2481	0.6287
5	Fdft1	0.1025	0.6962	−0.3079	0.5540
	Lipid associated transporters				
6	Abcg1	0.2871	0.3736	0.0414	0.9362
7	Abca1	−0.2284	0.4086	0.0487	0.9228
8	Abcg5	0.3795	0.2127	N/A	N/A
9	Abcg8	0.2989	0.3005	N/A	N/A
10	Abcb11	−0.2283	0.4070	**4.6214**	**0.0020**
11	Scarb1	0.0126	0.9610	0.2903	0.5731
12	Cd36	0.9179	0.0370	0.5995	0.2786
	Regulation of lipid homeostasis				
13	Apoa1	0.0452	0.8599	**5.0884**	**0.0014**
14	Apob	−0.0755	0.7696	**5.2388**	**0.0013**
15	Apoe	0.1057	0.6844	0.7665	0.1871
16	Mttp	−0.1132	0.6655	0.7328	0.2096
17	Lipc	−0.1058	0.6863	**5.3496**	**0.0014**
18	Pcsk9	0.0400	0.8782	**2.4986**	**0.0116**
19	Ldlr	0.0933	0.7205	0.2760	0.5918
20	Ceacam1	−0.1615	0.5463	−0.0182	0.9716
21	Cyp7a1	0.0446	0.8627	N/A	N/A
22	Cyp27a1	−0.2778	0.3279	0.6548	0.2494
23	Cyp8b1	−0.1789	0.5111	N/A	N/A
	Transcriptional factors in lipid metabolism				
24	Nr1h3	0.0957	0.7164	0.8362	0.1621
25	Nr1h2	−0.2196	0.4363	0.0276	0.9563
26	Nr1h4	0.2166	0.4320	0.2670	0.6078
27	Ppara	0.0929	0.7225	1.2696	0.0654
28	Pparg	0.0881	0.7576	0.5264	0.3352
29	Srebf1	−0.2218	0.4198	0.3281	0.5276
30	Srebf2	−0.2153	0.4383	−0.2076	0.6848

Note: Genes that were significantly up- or downregulated are highlighted in bold.

### Postnatal loss of *Dhcr24* resulted in viable mice

**Fig. 7:**
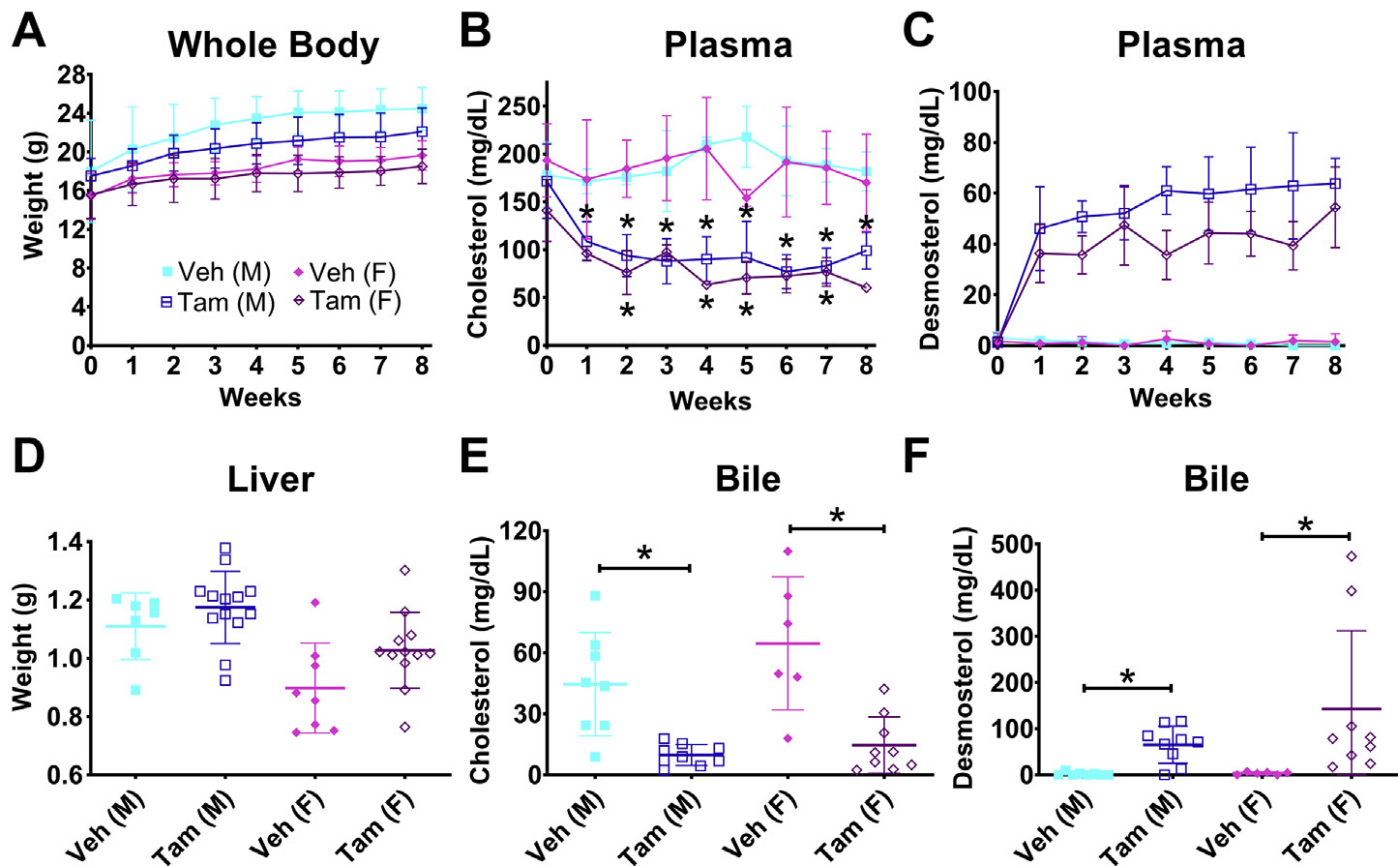
Characterization of mice lacking postnatal global expression of DHCR24. Body weights (A) and plasma cholesterol and desmosterol levels (B and C) were measured weekly for 8 weeks after injection of *Dhcr24*^flx/flx,Er-Cre^ mice with tamoxifen (Tam) or vehicle (Veh). Liver weights and biliary cholesterol and desmosterol levels were measured after euthanasia 8–9 weeks after injection (D–F). Body and liver weights did not differ significantly between Tam-treated mice and their sex-matched controls (A and D). As expected, plasma desmosterol was consistently elevated in the Tam-treated mice (C), but unlike LKO mice, cholesterol was also significantly lower in Tam-treated male and female *Dhcr24*^flx/flx,Er-Cre^ mice when compared with sex-matched Veh-treated controls (B). Biliary cholesterol and desmosterol levels mirrored the plasma level with elevated desmosterol and decreased cholesterol levels in Tam-treated mice (E and F). For body and liver weight measurements, N = 7 male Veh, N = 13 male Tam, N = 8 female Veh, and N = 12 female Tam mice, except in wk 5 where N = 7 for female Veh mice for body weight measurements. For plasma cholesterol and desmosterol measurements, N = 2 male Veh, N = 6 male Tam, N = 4 female Veh, and N = 3 female Tam mice, except in weeks 4 and 6 when N = 5 and in week 8 when N = 3 for male Tam mice, and in weeks 3 and 8 when N = 2 for female Tam mice. For biliary cholesterol and desmosterol measurements, N = 8 male Veh, N = 9 male Tam, N = 6 female Veh, and N = 9 female Tam mice. Males are represented with squares, and females with diamonds; closed symbols indicate CTL mice, and open symbols LKO mice. Bars denote mean ± 1 SD. (∗*P* < 0.05) Comparisons were performed only with sex-matched controls.

**Fig. 8:**
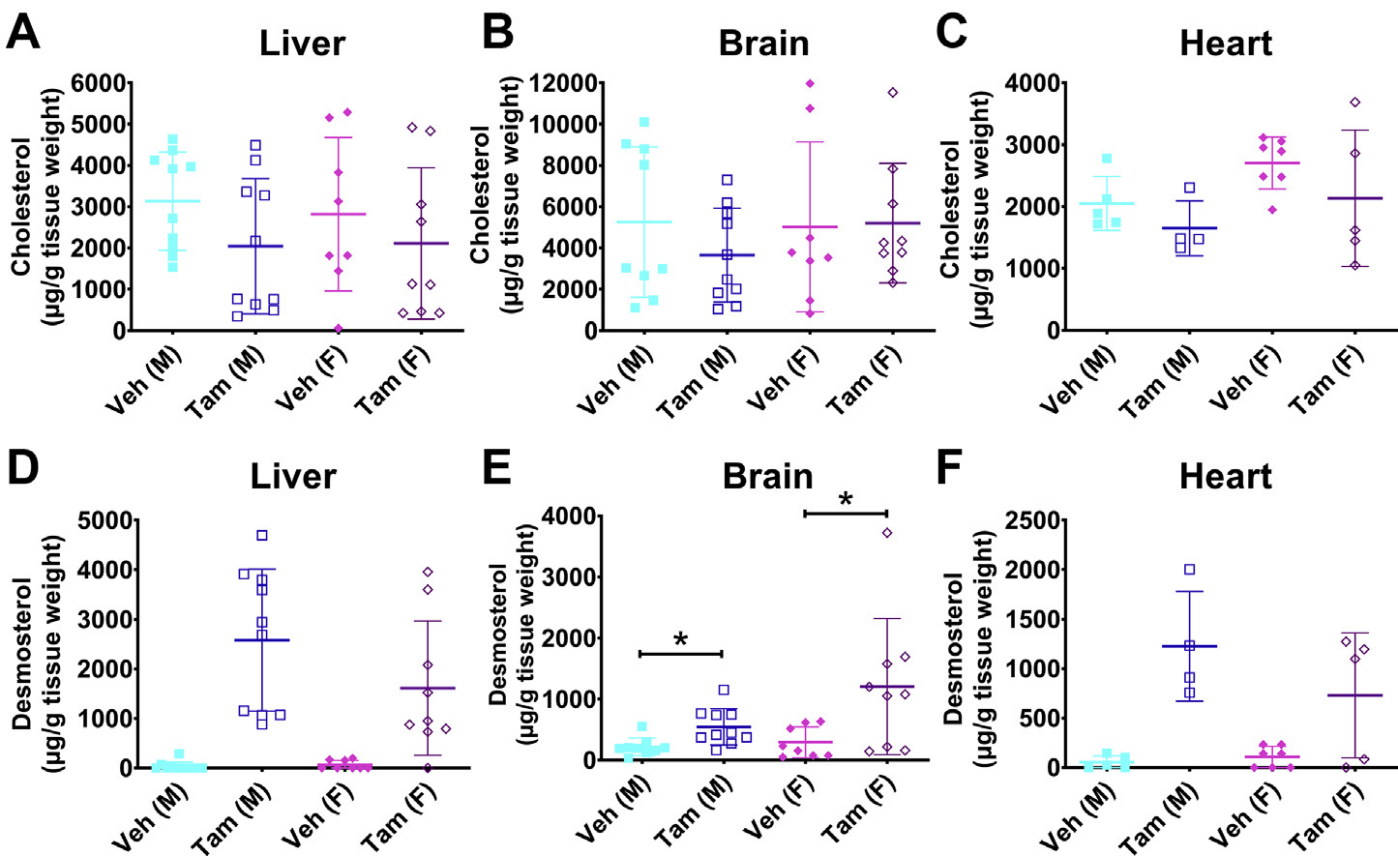
Effect of postnatal global *Dhc24* gene deletion on tissue sterol content. Cholesterol and desmosterol levels were measured in livers (A and D), brains (B and E), and hearts (C and F) of *Dhcr24*^flx/flx,Er-Cre^ mice harvested 8–9 weeks after injection with tamoxifen (Tam) or vehicle (Veh). As expected, after tamoxifen administration, desmosterol levels were dramatically increased in all three tissues (D–F). It is surprising that, despite the reduction in circulatory cholesterol, there was no reduction in the cholesterol content of tamoxifen-treated liver (A), brain (B), or heart (C). For liver measurements, N = 11 male Veh, N = 10 male Tam, N = 8 female Veh, and N = 9 female Tam mice; for brain measurements, N = 9 male Veh, N = 10 male Tam, N = 8 female Veh, and N = 9 female Tam mice. For heart measurements, N = 5 male Veh, N = 4 male Tam, N = 7 female Veh, and N = 5 female Tam mice. Males are represented with squares, and females with diamonds; closed symbols indicate CTL mice, and open symbols LKO mice. Bars denote mean ± 1 SD. Statistical testing was performed only to sex-matched controls, ∗*P* < 0.05.

Since embryonic global loss of *Dhcr24* results in neonatal death of homozygous pups within 10 h of birth [(18) and unpublished observations], we investigated if postnatal global deletion of *Dhcr24* would be viable using a tamoxifen-inducible cre line. *Dhcr24*^flx/flx,Er-Cre^ pups were viable after birth and had no obvious difference in appearance, mortality, or fertility (unpublished data). At 4–5 weeks of age, we injected tamoxifen or oil (vehicle) in matched littermates and tracked their weights and plasma sterol profiles (Fig. 7). Deletion of *Dhcr24* was confirmed by PCR of genomic DNA isolated from liver (supplemental Fig. S1). There were no significant differences noted in the growth curves between vehicle or tamoxifen-treated mice (Fig. 7A). Liver (Fig. 7D) and spleen (supplemental Fig. S2B) weights also remained similar between vehicle and tamoxifen-treated mice. In tamoxifen-treated mice, there was an early decline and stable decrease in plasma cholesterol (Fig. 7B) with a dramatic and very large increase in plasma desmosterol (Fig. 7C). Liver, brain, and heart tissue cholesterol levels remained comparable between vehicle and tamoxifen-treated mice (Fig. 8A–C) with significantly elevated levels of desmosterol in these tissues (Fig. 8D–F), as well as in bile (Fig. 7F). In contrast to *Dhcr24*^flx/flx,Alb-Cre^ mice, where levels of circulatory cholesterol were normal, *Dhcr24*^flx/flx,Er-Cre^ mice possessed markedly lower plasma (Fig. 7B) and biliary cholesterol (Fig. 7E) with a maximal reduction down to 50% of the levels found in controls. The mechanism for the decreased plasma cholesterol is unclear, as the cholesterol content of liver, brain, and heart were not markedly different between tamoxifen and vehicle mice (Fig. 8A–C).

Although the presence of desmosterol was not initially obvious, after we performed sterol analyses, we noted that some vehicle-injected mice had detectable desmosterol levels. Although very low, these levels are still comparatively high, as wild-type mice and *Dhcr24*^flx/flx^ mice had undetectable desmosterol levels under our assay conditions. At sacrifice, 8–9 weeks post tamoxifen injection, PCR analyses of both male and female liver samples confirmed that tamoxifen-induced tissues showed distinct *Dhcr24* gene deletion (supplemental Fig. S1). Unfortunately, vehicle-injected mice showed variable levels of gene deletion (presence of both WT and KO bands) confirming leaky ER-Cre activity (supplemental Fig. S1). Although the vehicle-injected mice are therefore not ideal controls because of the endogenous leakiness of this particular ER-Cre driver (and we caution against its use), these data remain informative; tamoxifen-injected mice showed dramatic elevations in desmosterol yet continued to show no major phenotypic ill-health.

## Discussion

We describe here the creation of a *Dhcr24* conditional knockout mouse model and verified its functionality by generating and characterizing an LKO. Although the first report of a global *Dhcr24* knockout proposed that a “cholesterol-free” mouse was possible (17), subsequent characterizations of this line with further breeding showed no survival of global *Dhcr24* KO pups beyond 24 h and lethality was likely caused by a lethal dermopathy with transepithelial water loss (18). Our initial attempts reproduced the perinatal lethality in *Dhcr24* homozygous null mice (unpublished observations). This prompted us to pursue conditional KO models in order to explore the role of *Dhcr24* in embryonic development. *Dhcr24*^flx/flx^ mice were developed and validated using cre-mediated deletion in liver, as well as postnatal global loss using tamoxifen-inducible ER-Cre. Both the liver-specific and the postnatal global loss of *Dhcr24* mice were remarkably normal and showed comparable growth curves and preserved fertility and manifested no overt phenotype. This suggests that almost all the phenotypes observed with humans with desmosterolosis may be as a result of embryonic loss of DHCR24 activity and that loss of DHCR24 postnatally may not be as detrimental; loss of *Dhcr24* in mice using a globally expressed ER-cre driver resulted in the absence of endogenous cholesterol synthesis and a dramatic elevation in desmosterol levels but was well tolerated.

Cholesterol homeostasis in mammals is a complex and tightly regulated process that involves a proper balance between its endogenous synthesis, cellular uptake, and efflux. Inherited post-squalene cholesterol biosynthetic pathway disorders show distinct patterns of precursor accumulation and variable tissue cholesterol reductions, depending on the severity of enzymatic impairments (31, 32). Therefore, it is difficult to determine whether excessive accumulation of sterol precursors or a decrease in cellular cholesterol or some combination of these is responsible for the pathogenesis of disease phenotypes (31, 32). The liver was chosen as a tissue of interest for targeted deletion of *Dhcr24* owing to its primary role in the maintenance of systemic cholesterol homeostasis (33). An accumulation of excess desmosterol in the livers but not in the brains of *Dhcr24*^flx/flx,Alb-Cre^ mice confirms the liver-specific knockout of *Dhcr24*. The maintenance of elevated levels of plasma desmosterol in *Dhcr24*^flx/flx,Alb-Cre^ mice indicated that there was secretion of hepatic desmosterol into the circulation (34), and the increased desmosterol in bile and stool of *Dhcr24*^flx/flx,Alb-Cre^ male mice suggested that desmosterol can use the hepatobiliary system for excretion from the body. Nevertheless, the ability of *Dhcr24*^flx/flx,Alb-Cre^ mice to have a mostly normal phenotype despite accumulation of circulatory and tissue desmosterol in the liver suggests that elevated desmosterol and a loss of DHCR24 enzyme activity are well tolerated. Elevated levels of lanosterol in the liver, when compared with control mice, indicated limited involvement of the Kandutsch-Russell pathway in the liver and predominant use of the Bloch pathway. This is expected as DHCR24 catalyzes the first step of entry into the Kandutsch-Russell pathway and the last step of the Bloch pathway. Of interest, these results suggest also that control animals had minimal or very efficient utilization of the Kandutsch-Russell pathway, since there was little accumulation of intermediates from this pathway, although more sensitive techniques (such as LC-MS/MS) to detect subtle elevations were not employed. Lanosterol, which is an early intermediate in post-squalene biosynthesis, was upregulated in both male and female *Dhcr24*^flx/flx,Alb-Cre^ mice, although these mice differed in liver cholesterol levels, suggesting additional sex-specific regulation of sterol homeostasis.

Liver X receptors (LXRs) are nuclear proteins and a member of receptor family transcription factors that are reported to upregulate genes involved in cholesterol efflux, thereby participating in cholesterol homeostasis (35). Two important LXR-regulated genes involved in cholesterol efflux are ATP-binding cassette transporter-A1 (*Abca1*) and ATP Binding Cassette Subfamily G Member 1 (*Abcg1*) (35). Earlier studies have suggested that desmosterol, zymosterol, and various oxysterols act as endogenous ligands for activation of these nuclear proteins (LXR) and through this mechanism participate in glucose and lipid homeostasis, steroidogenesis, and immunity (30). However, our data did not show significant upregulation of *Nr1h3* and LXR target gene expression in livers of *Dhcr24*^flx/flx,Alb-Cre^ mice despite elevated circulatory and tissue levels of desmosterol. This is consistent with a previously published study by Muse *et al.* (36) that found that desmosterol had almost no activity in hepatocytes.

Notably missing from the list of differentially expressed genes in RNA-Seq analysis were genes involved in cholesterol biosynthesis. It seems that the loss of hepatic *Dhcr24* did not result in upregulation of the cholesterol biosynthesis pathway. The accumulation of desmosterol may compensate for the loss of cholesterol, resulting in no detected change in the total sterol content sensed by the hepatocyte, or the liver may receive so much cholesterol from the periphery that unless that is altered, hepatic sterol balance may not be impacted by loss of DHCR24. Postnatal global deletion of *Dhcr24* showed that plasma cholesterol fell significantly, while total plasma sterol levels seemed unaltered, as the decrease in cholesterol was matched by the significant rise in desmosterol.

Finally, we also asked if a global deletion of *Dhcr24* was lethal. Although vehicle-injected *Dhcr24*^flx/flx,Er-Cre^ mice showed leaky expression of Cre recombinase, the tamoxifen-treated mice are nevertheless informative; almost complete whole-body loss of cholesterol synthesis is compatible with life; growth parameters seem relatively normal and the accumulation of pharmacological amounts of desmosterol in the blood (∼60 mg/dl) seems to be well tolerated. This result is expected given that there are some surviving adult patients with desmosterolosis and also because whole-body inhibition of cholesterol synthesis using Triparanol had been used on humans as a treatment of elevated cholesterol in the last century (37, 38). Future studies, using a more specific ER-cre, are planned to examine if there is a long-term phenotype, especially on nervous system function as the blood-brain barrier is a limitation for dietary cholesterol entry.

In conclusion, we demonstrated that *Dhcr24* is not necessary for survival after fetal development. Insights derived from the pathways identified by RNA-Seq analysis suggest many potential mechanisms by which desmosterol accumulation may be clinically important. However, expression of genes involved in cholesterol synthesis and metabolism seem not to be affected by the deletion of *Dhcr24*, although direct measurements of sterol synthesis rates were not performed. *Dhcr24*^*flx*/flx^ mice can serve as a valuable tool to investigate the role of post-squalene synthesis in development, as well as study the long-term effects of *Dhcr24* deletion on mammalian physiology.

### Data availability

The RNA-Seq data have been deposited with the NCBI Gene Expression Omnibus (GEO) database (#GSE146524), and all primary raw data are available from the corresponding author.

## Conflict of interest

The authors declare that they have no conflicts of interest with the contents of this article.
