# Supplementary material for: Generation and validation of a conditional knockout mouse model for desmosterolosis
Source: J Lipid Res. 2021 Jan 30;62:100028. doi: 10.1016/j.jlr.2021.100028 (PMC7933790; doi:10.1016/j.jlr.2021.100028)
Supplement: Supplemental Tables S1 & S2 and Figures S1 to S4 [file mmc1.docx]

**SUPPLEMENTAL FIGURES:**

**Generation and validation of a conditional knockout mouse model for desmosterolosis**

Babunageswararao Kanuri^1^, Vincent Fong^1^, Sithara Raju Ponny^2#^, Ranjuna Weerasekera^3#^, Kirthy Pulakanti^4^, Kriya Patel^5#^, Roman Tyshynsky^5#^, Shailendra B. Patel^1*^

1. Division of Endocrinology, Diabetes and Metabolism, University of Cincinnati, Cincinnati, OH, USA
2. Division of Human Genetics, Cincinnati Children’s Hospital Medical Center, Cincinnati, OH, USA
3. Division of Endocrinology, Medical College of Wisconsin, Milwaukee, WI, USA
4. Blood Research Institute, Versiti, Milwaukee, WI, USA
5. Clement J. Zablocki Veterans Affairs Medical Center, Milwaukee, WI, USA

*To whom correspondence should be addressed.

e-mail: [sbpatel@uc.edu](mailto:sbpatel@uc.edu)

**Supplemental Table S1. Pathways enriched by significantly downregulated genes**

| **ID** | **Term** | **C^*^** | **P Value** | **Genes** | **q-value**  **Bonferroni** |
| --- | --- | --- | --- | --- | --- |
| **Female *Dhcr24*^flx/flx,Alb-Cre^ liver** | | | | | |
| SMP00130 | Steroidogenesis | 4 | 2.18E-07 | CYP11A1, CYP11B1, CYP21A2, HSD3B1 | 1.62E-04 |
| MAP00140_C21 | Steroid hormone metabolism | 4 | 6.45E-07 | CYP11A1, CYP11B1, CYP21A2, HSD3B1 | 4.81E-04 |
| 1270046 | Metabolism of steroid hormones | 5 | 3.98E-06 | CYP11A1, CYP11B1, CYP21A2, STAR, HSD3B1 | 2.97E-03 |
| 1270048 | Glucocorticoid biosynthesis | 3 | 3.50E-05 | CYP11B1, CYP21A2, HSD3B1 | 2.61E-02 |
| 545305 | Mineralocorticoid biosynthesis | 2 | 1.72E-04 | CYP11B1, CYP21A2 | 1.28E-01 |
| **Female *Dhcr24*^flx/flx,Alb-Cre^ adrenal gland** | | | | | |
| 1269923 | Transport of glucose and other sugars, bile salts and organic acids, metal ions and amine compounds | 6 | 1.82E-05 | SLC5A7, RSC1A1, SLC6A18, EMB, RHAG, SLC6A2 | 7.05E-03 |
| 1269456 | p75NTR regulates axonogenesis | 3 | 2.81E-05 | NGF, NGFR, OMG | 8.44E-03 |
| 1269446 | NFG and proNGF binds to p75NTR | 2 | 2.70E-05 | NGF, NGFR | 1.05E-02 |
| 1269455 | Ceramide signaling | 2 | 8.03E-05 | NGF, NGFR | 3.11E-02 |
| 1269457 | Axonal growth stimulation | 2 | 1.60E-04 | NGF, NGFR | 6.21E-02 |

* refers to hit count in query list

**Supplemental Table S2. Pathways enriched by significantly upregulated genes**

| **ID** | **Term** | **C^*^** | **P Value** | **Genes** | **q-value**  **Bonferroni** |
| --- | --- | --- | --- | --- | --- |
| **Female *Dhcr24*^flx/flx,Alb-Cre^ liver** | | | | | |
| M6487 | Platelet Amyloid Precursor Protein Pathway | 4 | 9.85E-06 | PLAT, PLAU, COL4A3, COL4A4 | 7.93E-03 |
| M5889 | Ensemble of genes encoding extracellular matrix and extracellular matrix-associated proteins | 24 | 1.12E-04 | PDGFB, GDF6, MFAP4, CCL4, PLAT, PLAU, CLEC10A, CCL22, MMP12, COL4A3, COL4A4, CXCL14, VCAN, EMCN, SEMA3E, BDNF, PTN, FGF21, VWA3B, ADAMTSL1, ECM2, ESM1, LAMC2, VWA7 | 9.00E-02 |
| 1270207 | Phase II conjugation | 7 | 1.44E-04 | TPMT, NAT1, UGT1A8, SULT1E1, SULT2A1, GSTA1, GSTA2 | 1.16E-01 |
| **Female *Dhcr24*^flx/flx,Alb-Cre^ adrenal gland** | | | | | |
| 83073 | Complement and coagulation cascades | 30 | 4.14E-30 | MBL2, VSIG4, F2, F5, F7, F9, F10, F11, F12, F13B, FGA, FGB, FGG, PLG, SERPINF2, VTN, CPB2, CFI, SERPINC1, PROC, MASP2, CFB, C5, C8A, C8B, C8G, C9, KLKB1, KNG1, SERPIND1 | 4.45E-27 |
| SMP00274 | Heparin Pathway | 14 | 9.27E-21 | F2, F7, F9, F10, F11, F12, F13B, FGA, FGB, FGG, PLG, SERPINC1, KLKB1, KNG1 | 9.97E-18 |
| SMP00273 | Fondaparinux Pathway | 14 | 9.27E-21 | F2, F7, F9, F10, F11, F12, F13B, FGA, FGB, FGG, PLG, SERPINC1, KLKB1, KNG1 | 9.97E-18 |
| SMP00275 | Ardeparin Pathway | 14 | 9.27E-21 | F2, F7, F9, F10, F11, F12, F13B, FGA, FGB, FGG, PLG, SERPINC1, KLKB1, KNG1 | 9.97E-18 |
| SMP00272 | Enoxaparin Pathway | 14 | 9.27E-21 | F2, F7, F9, F10, F11, F12, F13B, FGA, FGB, FGG, PLG, SERPINC1, KLKB1, KNG1 | 9.97E-18 |

* refers to hit count in query list


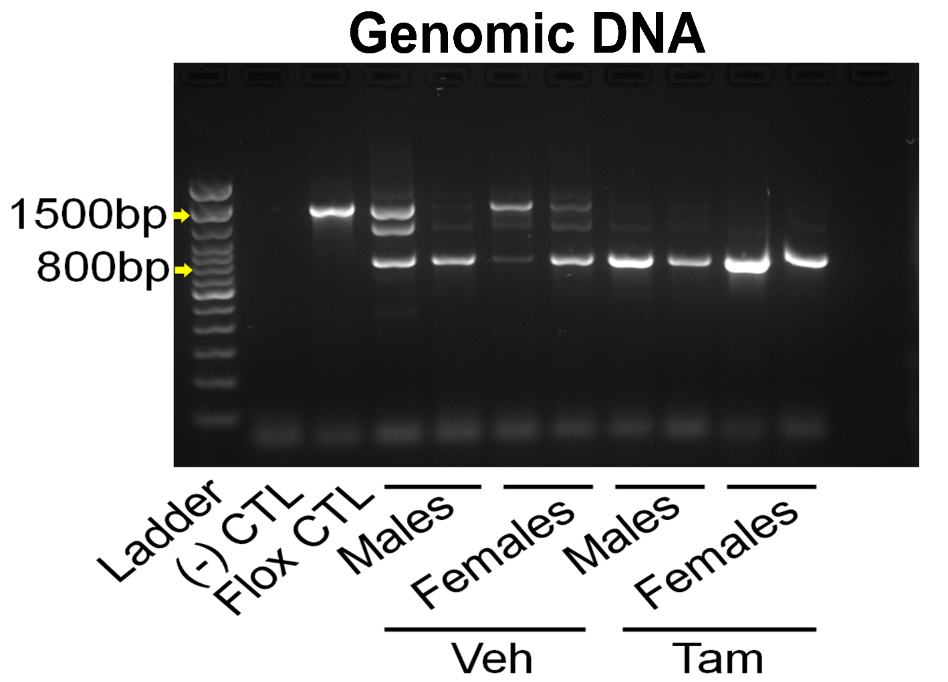


**Supplemental Fig. S1. Presence of leakiness in the liver samples of vehicle treated *Dhcr24*^flx/flx,Er-Cre^ mice.** PCR targeting *Dhcr24* exon 3 of genomic DNA isolated from the livers of vehicle or tamoxifen treated mice were run on 1.5% agarose gel and visualized. Vehicle (Veh) treated *Dhcr24*^flx/flx,Er-Cre^ mice showed a clearly visible floxed WT band at ~1400bp and a gene deletion band at ~800bp, while the tamoxifen (Tam) treated samples showed only a deletion band.


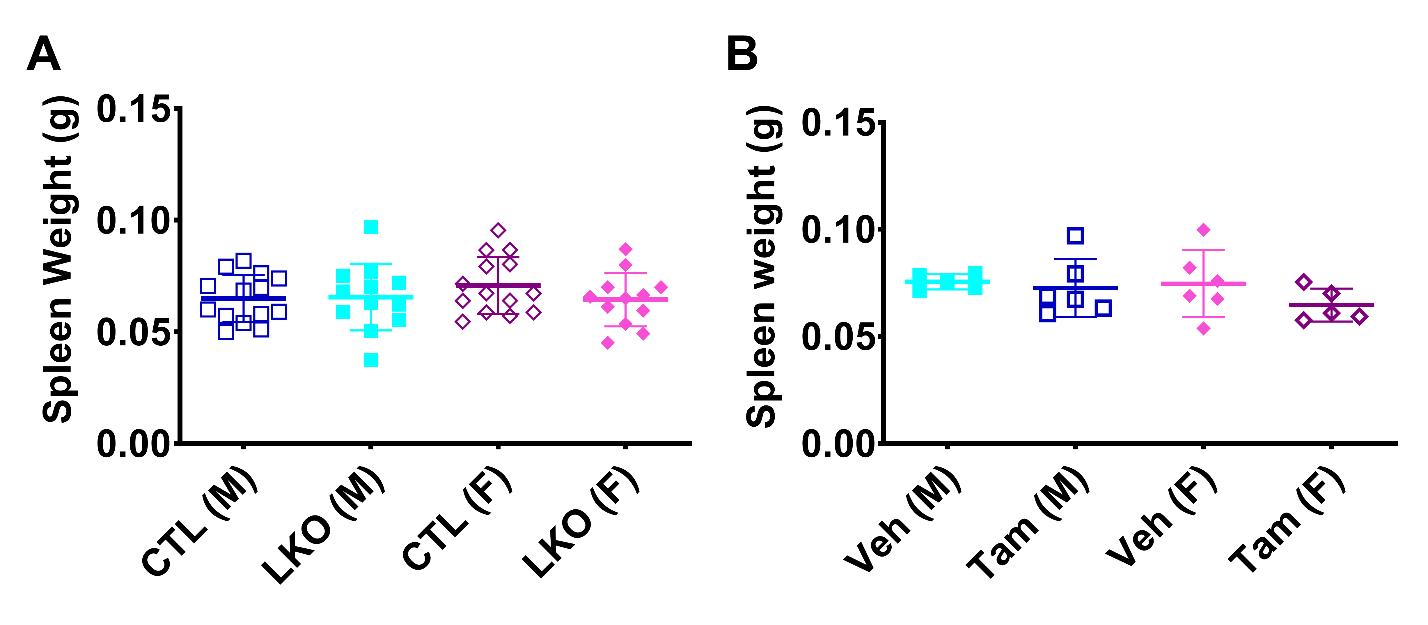


**Supplemental Fig. S2. Spleen weights.** Spleen weights were measured in tissues collected in CTL and LKO mice (panel A) at age 10-12wk old or from *Dhcr24*^flx/flx,Er-Cre^ mice harvested 8-9weeks after injection with tamoxifen (Tam) or vehicle (Veh). N=9 male CTL, N=7 male LKO, N=10 female CTL, N=10 female LKO mice, N=7 male Veh, N=13 male Tam, N=8 female Veh, and N=12 female Tam mice. Males are represented with squares, and females with diamonds; closed symbols indicate CTL mice, and open symbols LKO mice. Bars denote mean ± 1SD.

**
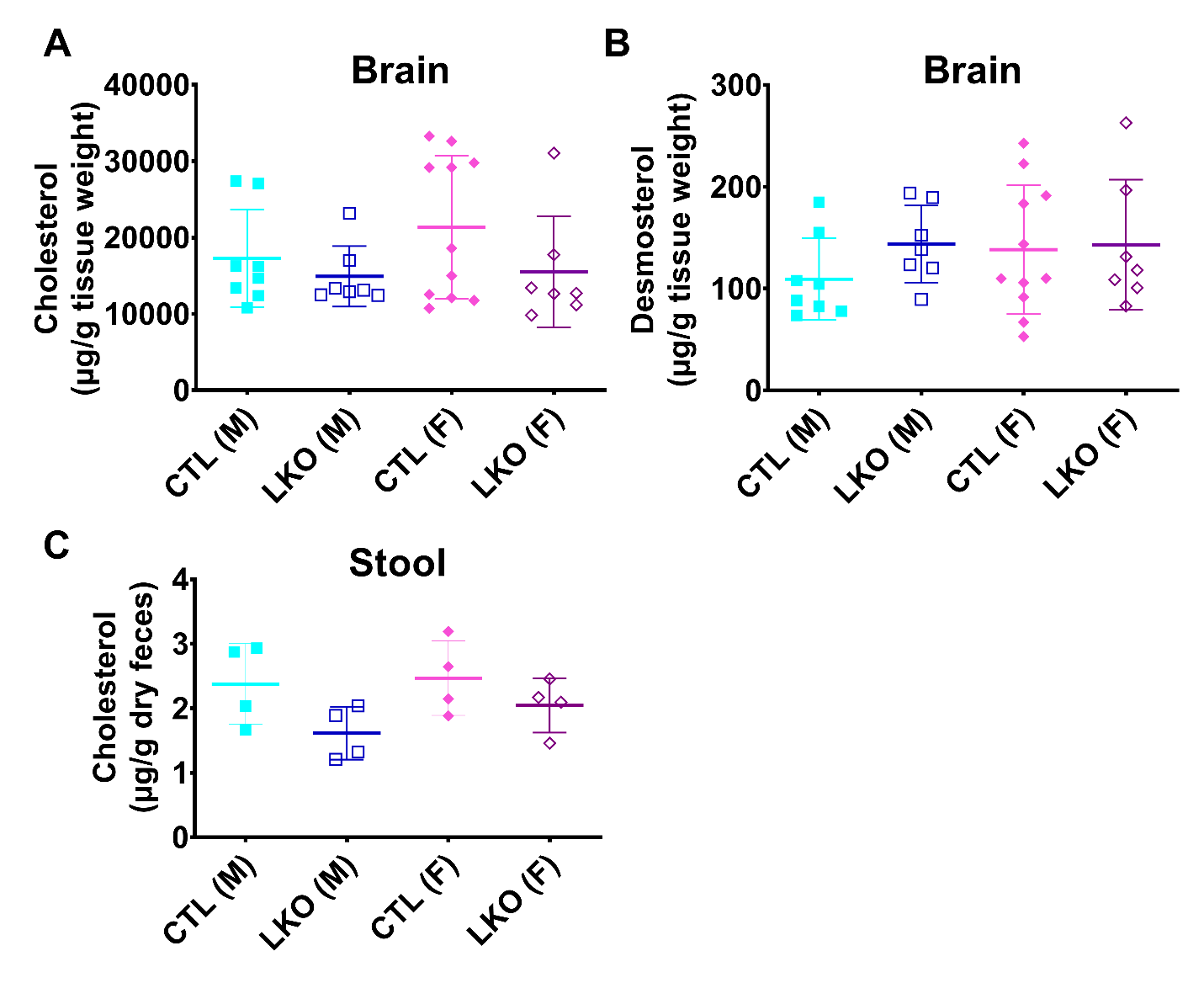
**

**Supplemental Fig. S3:** **Brain cholesterol and desmosterol, and stool cholesterol in *Dhcr24*^flx/flx,Alb-Cre^ mice.** Cholesterol and desmosterol levels in brains (panels A&B), and cholesterol levels in stool (panel C) of CTL and LKO mice are shown. There were no significant differences found in these parameters between LKO mice and their sex-matched controls. Brains were collected from mice after euthanasia at age 10-12 wk. For brain measurements, n=8 male CTL, 7 male LKO, 11 female CTL, 7 female LKO mice; for stool measurements, n=4 in all groups. Males are represented with squares, and females with diamonds; closed symbols indicate CTL mice, and open symbols LKO mice. Bars denote mean ± 1SD.


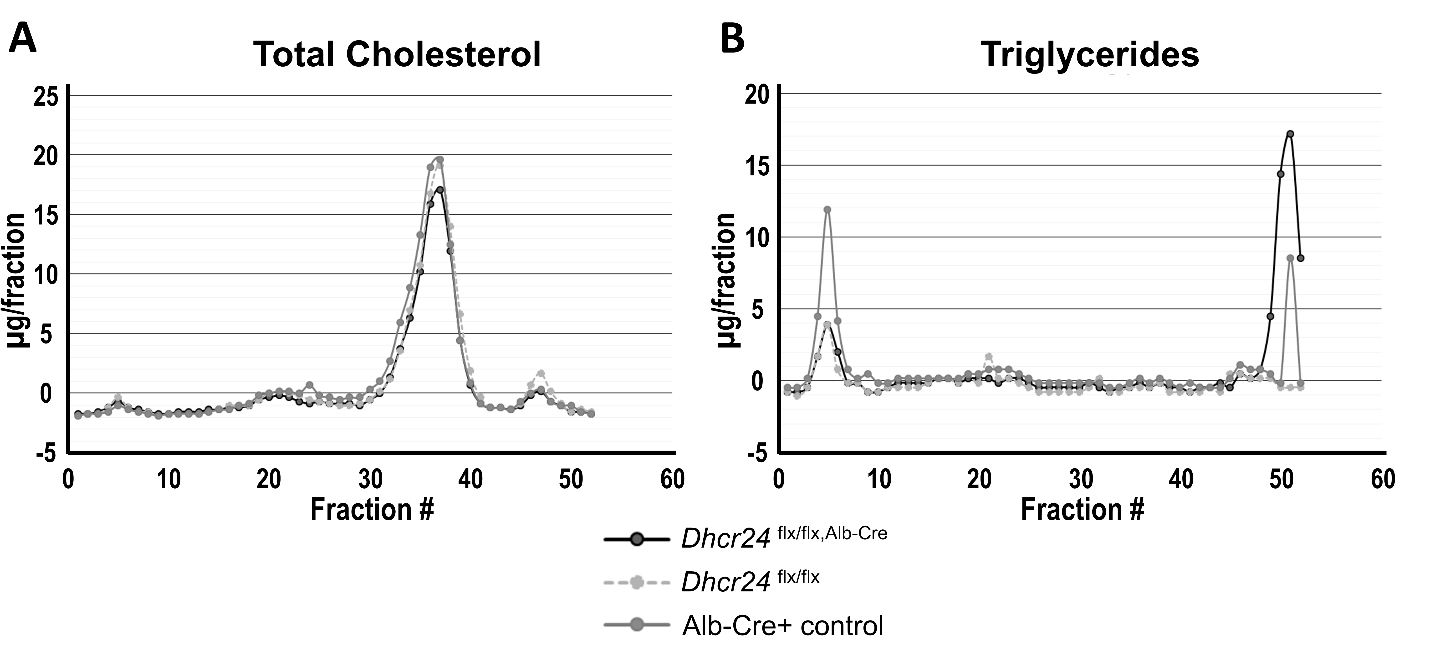


**Supplemental Fig. S4. FPLC total cholesterol and triglyceride lipoprotein profiles.** Pooled plasma samples were run on the Akta pure FPLC, and a total of 52 fractions each were collected and analyzed for total cholesterol and triglycerides. Lipoprotein profiles were similar between *Dhcr24*^flx/flx,Alb-Cre^, *Dhcr24*^flx/flx^ and Alb-Cre+ controls. The ‘triglyceride’ peaks in fractions 50 onwards in panel B likely represent non-lipoprotein glycerol.


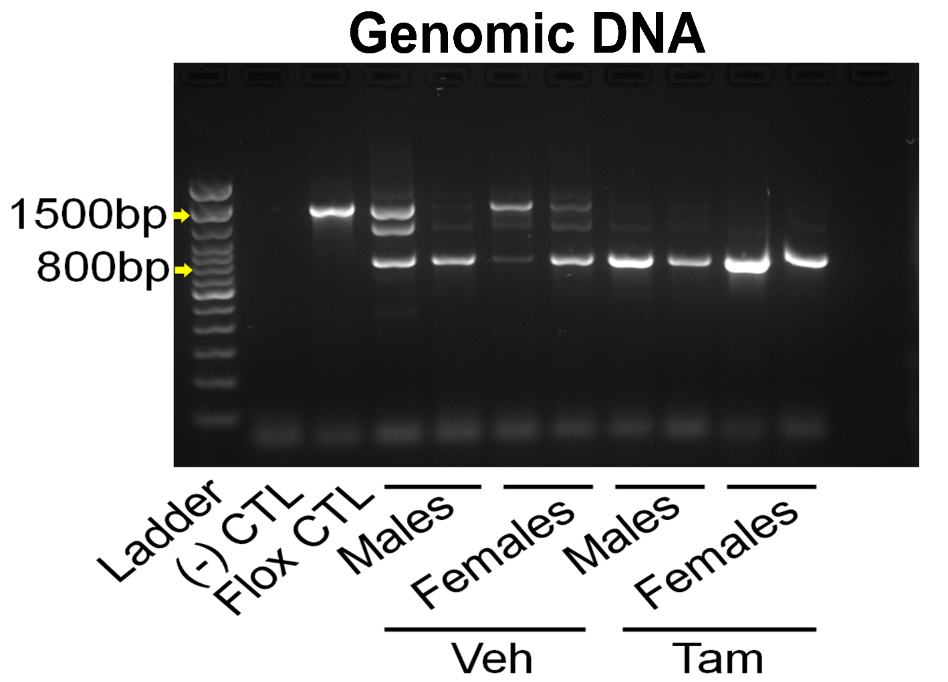


**Supplemental Fig. S1. Presence of leakiness in the liver samples of vehicle treated *Dhcr24*^flx/flx,Er-Cre^ mice.** PCR targeting *Dhcr24* exon 3 of genomic DNA isolated from the livers of vehicle or tamoxifen treated mice were run on 1.5% agarose gel and visualized. Vehicle (Veh) treated *Dhcr24*^flx/flx,Er-Cre^ mice showed a clearly visible floxed WT band at ~1400bp and a gene deletion band at ~800bp, while the tamoxifen (Tam) treated samples showed only a deletion band.
